# Diagnostic Underuse and Antimicrobial Resistance Patterns Among Hospitalized Children in a National Referral Hospital in Kenya: A Five-Year Retrospective Study

**DOI:** 10.3390/antibiotics14090872

**Published:** 2025-08-29

**Authors:** Veronicah M. Chuchu, Teresa Ita, Irene Inwani, Julius Oyugi, S. M. Thumbi, Sylvia Omulo

**Affiliations:** 1 Department of Medical Microbiology, University of Nairobi, Nairobi P.O. Box 19676-00202, Kenya; 2 Washington State University Global Health-Kenya, Nairobi P.O. Box 72938-00200, Kenya; teresa.ita@wsu.edu; 3 Center for Epidemiological Modelling and Analysis, University of Nairobi, Nairobi P.O. Box 19676-00202, Kenya; thumbi.mwangi@wsu.edu; 4 FIND, 1218 Geneza, Switzerland; 5 Commonwealth Pharmacists Association, 66–68 East Smithfield, London E1W 1AW, UK; 6 Kenyatta National Hospital, Nairobi P.O. Box 20723-00202, Kenya; iinwani@yahoo.com; 7 Department of Paediatrics, University of Nairobi, Nairobi P.O. Box 19676-00202, Kenya; 8 University of Nairobi Institute of Tropical and Infectious Diseases, Nairobi P.O. Box 19676-00202, Kenya; oyugi.otieno@uonbi.ac.ke; 9 Institute of Immunology and Infection Research, University of Edinburgh, Edinburgh EH9 3FL, UK; 10 Paul G. Allen School for Global Health, Washington State University, Pullman, WA 99164-7090, USA

**Keywords:** antimicrobial resistance, multidrug-resistant organisms, diagnostic utilization, pediatric infections, pediatric, East Africa

## Abstract

**Background:** Antimicrobial resistance (AMR) is a growing global health threat, with children in low- and middle-income countries bearing a disproportionate burden. Data on resistance patterns and diagnostic practices in pediatric populations remain limited. This study evaluated diagnostic utilization and AMR among children hospitalized with bacterial infections at a national referral hospital in Kenya. **Methods:** We conducted a retrospective cohort study of pediatric inpatients (0–12 years) admitted with bacterial infections between 2017 and 2021. Patient records were identified using ICD-10 codes and reviewed for diagnostic testing and antimicrobial susceptibility. Descriptive statistics were conducted to show infection counts, diagnostic testing, and resistance outcomes. **Results:** Among 1608 patients, 1009/1608 (63%) were infants under one year. Culture was conducted in 640/1608 (40%) and antimicrobial sensitivity testing in 111/640 (17%) patients. Gastroenteritis (46%) was the most common infection and blood the most frequently collected specimen (31%). Of 1039 cultured specimens, 896/1039 (86%) showed no growth. The most commonly isolated organisms were *Klebsiella pneumoniae* 19/128 (15%), *Staphylococcus epidermidis* (13%, 17/128), and *Enterococcus faecium* (13%, 16/128). Notably, *K. pneumoniae* showed 100% resistance to third-generation cephalosporins, suggestive of ESBL production. Among the tested samples, 92/128 (72%) had MDROs, and 26/92 (28%) were extensively drug-resistant (XDR). Among the patients tested, 84/111 (76%) had MDROs, of which 25/84 (30%) were XDR. Children under 5 years had higher odds (OR = 5.84, 95% CI: 1.17-38.21) of having MDRO infections, as well as those with multiple admissions (OR = 3.77, 95% CI: 1.06–20.34). Further, increasing age was inversely associated with MDRO presence. The odds of MDRO infection decreased by 24% for every year increase in age (aOR = 0.76; 95% CI: 0.60–0.93; *p* = 0.006). **Conclusions:** The findings highlight the limited diagnostic use and a high burden of MDROs and XDR infections in hospitalized children. Strengthening diagnostic capacity and pediatric antimicrobial stewardship is urgently needed in such settings.

## 1. Introduction

Antimicrobial resistance (AMR) poses a significant global health threat, particularly impacting vulnerable populations such as hospitalized children in low- and middle-income countries (LMICs). The World Health Organization (WHO) has identified AMR as one of the top ten global public health threats facing humanity, emphasizing its potential to undermine advancements in healthcare, food security, and life expectancy [1].

In sub-Saharan Africa, the burden of AMR is especially pronounced with 27.3 deaths per 100,000 population being reported in 2019 only in Western sub-Saharan Africa, largely due to limited diagnostic capacity, unregulated antibiotic use, weak surveillance systems, and a high burden of infectious diseases [2,3]. Studies have highlighted that among neonates, Gram-negative organisms are the predominant cause of early-onset sepsis, with a high prevalence of extended-spectrum β-lactamase-producing organisms. The high resistance rates to commonly used antibiotics such as ampicillin, gentamicin, and cefotaxime compromise the effectiveness of standard treatment protocols [4,5,6].

The overuse and misuse of antibiotics exacerbate the AMR crisis [7] with global antibiotic consumption being estimated to have increased by 35% between 2000 and 2010 [8] and by 65% between 2000 to 2015 [9]. In LMICs, the use of antibiotics that the WHO has designated critically important for human health increased by 165% between 2000 and 2015 [7]. Further, the estimated consumption of antibiotics by 2030 could increase by up to 200% if no policies change, these will be higher than the 2015 estimates of 41 billion defined daily doses [8]. Almost two tons of antibiotics are used every ten minutes around the world, all too often without a prescription or regulation [10]. These increases have been propelled by LMICs where the expanding utilization of antibiotics correlates with the growth of gross domestic product per capita [9]. Policies to enhance antibiotic consumption and reduce resistance can be informed by data on the consumption patterns of antibiotics over time across countries [9].

The high burden of communicable diseases in African countries leads to the extensive use of antimicrobials and subsequent resistance, with substantial financial, health, and societal implications [11]. The lack of diagnostic capacity and limited accessibility to reliable laboratory services means that many of these infections are treated without identifying the causative pathogens. This not only fails to effectively treat infections but also promotes the development and spread of resistant strains [12,13,14]. Low political prioritization is the key cause of poor access to diagnostics, with 47% of the global population being reported to have no access to diagnostics in 2021 [15]. In Kenya, the National Situation of AMR and consumption analysis report mapped 1037 laboratories, out of which only 64 reported the capacity for bacteriology testing [16]. Although the importance of diagnostics was demonstrated during the COVID-19 pandemic, their role in guiding the treatment of infections and curbing the emergence of AMR remains underrecognized [17].

Children are significant consumers of antibiotics [18,19] due to their high susceptibility to infectious diseases, immature immune systems, and challenges in diagnostics, especially in LMICs, leading to empirical antibiotic treatment. Consequently, AMR prevalence in this population, including multidrug-resistant organisms, is rising, making significant contributions to morbidity and mortality [20], prolonged hospital stays, and higher healthcare costs [4]. One in five deaths in children younger than five years has been reported to be caused by bacterial resistance [16]. In recent years, a few antibiotic agents have been developed specifically for use in children and newborns. Furthermore, those developed do not always adequately address the unique needs of the pediatric population for relevant bacterial priority pathogens [20]. With the rising antibiotic resistance, more effective antibiotics are fundamental in addressing AMR in children.

Addressing AMR requires a multifaceted approach, including the implementation of antimicrobial stewardship programs, investment in diagnostic infrastructure, and the development of updated treatment guidelines based on local resistance patterns [21,22]. However, the lack of specific data on resistance patterns, especially in the pediatric population, hinders the implementation of AMR interventions [18,23]. This study aims to evaluate antibiotic resistance patterns and diagnostic utilization among hospitalized children in a national referral hospital in Kenya. By analyzing the prevalence of AMR and utilization of diagnostics, this research seeks to inform clinical decision-making and policy development to mitigate the impact of AMR in the pediatric population.

## 2. Results

### 2.1. Patient Demographics

A total of 1608 pediatric patients were admitted with bacterial infections. Most patients (63%, 1009/1608) were under one year old. Over half of this cohort were males (57%, 923/1608), and more than one-third (38%, 608/1608) were referred from other healthcare facilities.

### 2.2. Infection Types

Among the 1608 patients, 638/1608 (40%) presented with more than one infection. Gastroenteritis was the most frequently documented infection, accounting for 1052/2287 (46%) of all reported infection events. This was followed by bacterial pneumonia (28%,639/2287), sepsis (12%,265/2287), and bacterial meningitis (11%, 239/2287). Urinary tract infections (UTIs) were comparatively uncommon, representing only 42/2287(2%) cases. Other infection types, including wound infections, pharyngitis, tracheitis, and tonsillitis, were infrequent, each accounting for less than 2% of cases (Supplementary Figure S1).

### 2.3. Diagnostic Utilization

#### 2.3.1. Culture Requests

Clinical isolates were cultured using standard bacteriological techniques. Identification of bacterial species and AST were performed using the VITEK2 automated system. Clinical and Laboratory Standards Institute (CLSI) M100 guidelines were used to interpret antibiotic susceptibility breakpoints.

Culture was conducted in 640/1608 (40%) patients. Of these, AST was documented in 111/640 (17%) cases. Culture and sensitivity tests were requested in a minority of cases across nearly all infection types. Notably, only 377/1051 (36%) patients with gastroenteritis, 259/639 (41%) with pneumonia, and 104/265(39%) with sepsis had a culture test requested, despite their high frequency. Meningitis had the highest diagnostic request rate (67%, 160/239), while culture testing was almost absent for tracheitis, pharyngitis, and wound infections (Supplementary Figure S1).

The majority of patients were admitted in 2021 (26%, 413/1608) and 2019 (22%, 347/1608). Comparing diagnostic utilization over the five years based on culture and AST request, cases in 2021 (49%, 204/413) and 2018 (45%, 125/279) had the highest demand as shown in Supplementary Figure S2.

#### 2.3.2. Sample Types and Culture Yield

**Figure 1:**
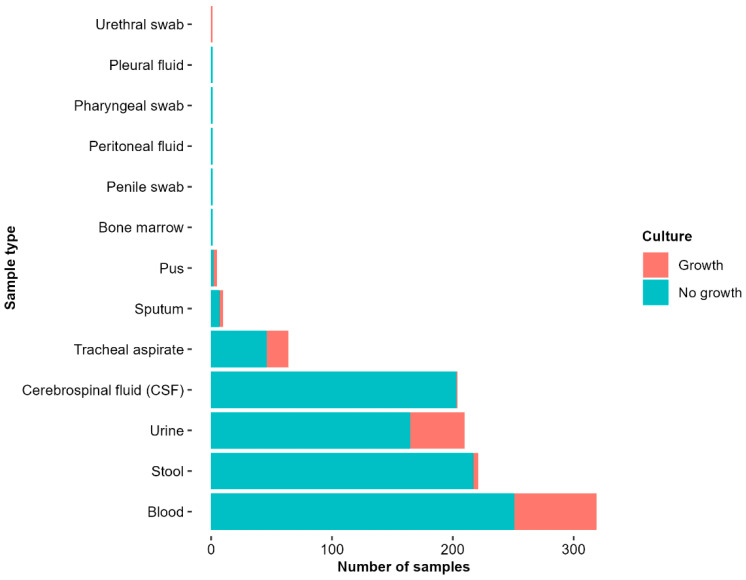
Culture results by sample type from pediatric inpatients (2017–2021). The bar chart presents the number of samples collected by specimen type and their corresponding culture outcomes (growth vs. no growth). Blood, stool, and urine were the most frequently collected samples, with the majority of the samples showing no growth.

In total, 1039 clinical samples were collected from the study population, with a median of 1 sample per patient (range: 1–8 samples). Blood accounted for 319/1039 (31%) of the total samples, stool for 221/1039 (21%), and urine and cerebrospinal fluid (CSF) for 210/1039 (20%) each. Of all samples cultured, 896/1039 (86%) had no bacterial growth, including 251/318 (79%) blood cultures, 217/221 (98%) stool cultures, 165/210 (79%) urine cultures, 203/204 (100%) CSF cultures, and 60/85 (71%) other cultures (Figure 1).

#### 2.3.3. Pathogen Identification and Antimicrobial Susceptibility Testing

**Figure 2:**
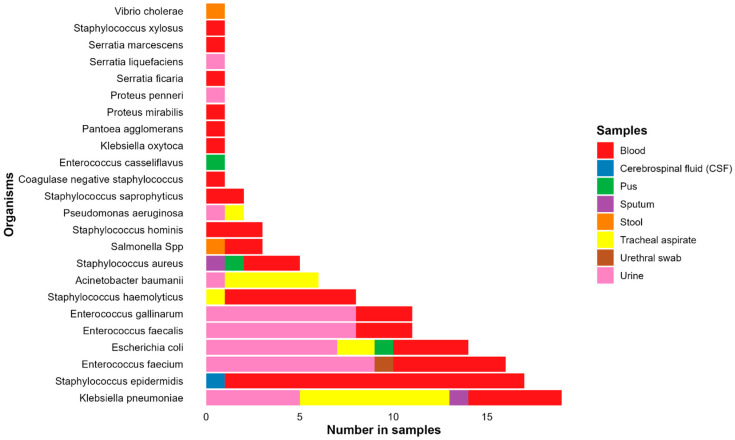
Common bacterial pathogens isolated from different sample types highlighting *Klebsiella pneumoniae*, *Staphylococcus epidermidis*, and *Enterococcus faecium* as the most identified pathogens.

Out of 143 samples with bacterial growth, AST was performed on 128 isolates (90%). Of these 128, *Klebsiella pneumoniae* was the most frequently identified organism, with 19 isolates (15%). This was followed by 17 *Staphylococcus epidermidis* (13%), 16 *Enterococcus faecium* (13%), 14 *Escherichia coli* (11%), and 11 (each) of *Enterococcus faecalis* and *Enterococcus gallinarum* (9% each). Other organisms identified in five or more cases included *Staphylococcus haemolyticus* (6%, 8 isolates), *Acinetobacter baumannii* (5%, 6 isolates), and *Staphylococcus aureus* (4%, 5 isolates) (Figure 2).

*K. pneumoniae* was more frequently isolated from tracheal aspirate samples (42.11%, 8/19 isolates) than from urine (26%, 5/19), blood (26%, 5/19), or sputum (5%, 1/19). Most (94%, 16/17) *S. epidermidis* isolates were recovered from blood, while *E. faecium* isolates were mostly recovered from urine (56%, 9/16) or blood (38%, 6/16). Of the 14 *E. coli* isolates, 7 were recovered from urine, 4 from blood, and the rest from other samples (Figure 2).

### 2.4. Antibiotic Resistance

**Figure 3:**
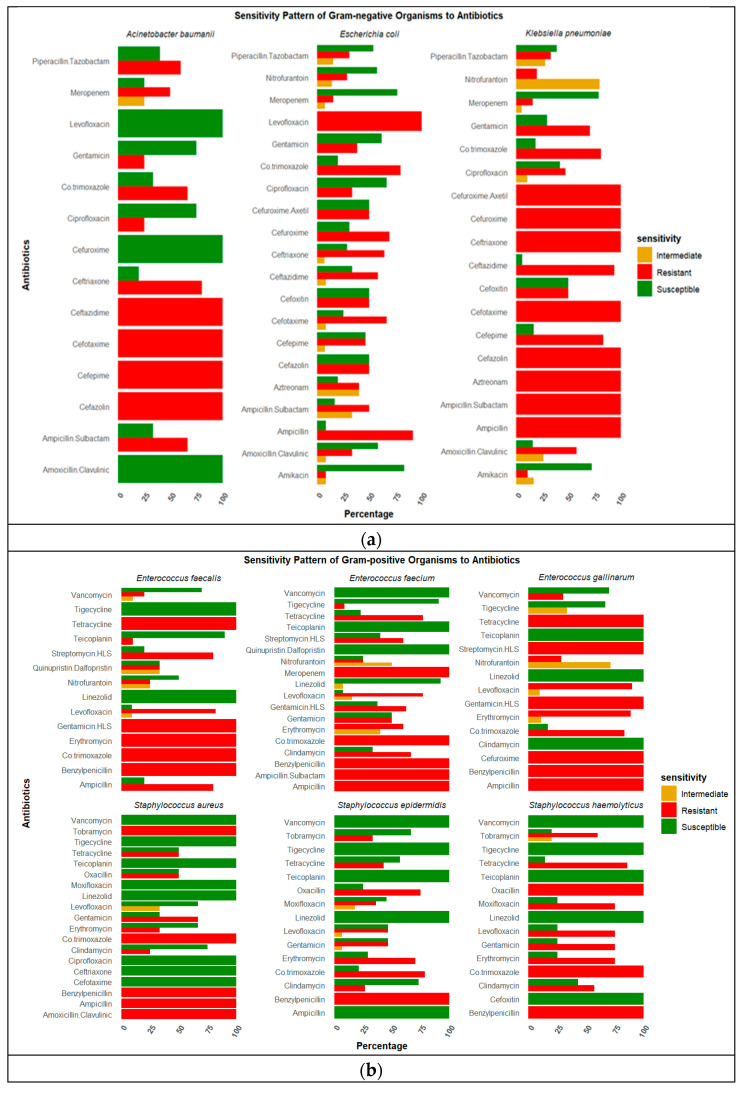
(**a**): The chart illustrates the antimicrobial sensitivity pattern for isolated Gram-negative pathogens including *Klebsiella pneumoniae, Escherichia coli*, and *Acinetobacter baumanii.* Green indicates susceptible, red resistant, and mustard intermediate. *Klebsiella pneumoniae* is showing resistance to most of the antibiotics tested. (**b**): The chart illustrates the antimicrobial sensitivity pattern of isolated Gram-positive pathogens including *Staphylococcus epidermidis, Enterococcus faecium*, and *Enterococcus faecalis*, among others. Green indicates susceptible, red resistant, and mustard intermediate. *E. faecium* is showing resistance to most of the antibiotics tested.

All *K. pneumoniae* isolates were resistant to ampicillin, cefazolin, and third-generation cephalosporins, regardless of specimen source. Notably, isolates from blood and tracheal aspirates showed near-complete resistance to beta-lactams and aminoglycosides, with retained susceptibility to meropenem and amikacin. Resistance to extended-spectrum beta-lactams and aztreonam was universal. Moderate susceptibility was observed to amikacin (72%, 13/18) and meropenem (83%, 15/18) (Figure 3a).

*E. coli* isolates were resistant to ampicillin, cefuroxime, third-generation cephalosporin, and amoxicillin clavulanic and were susceptible to amikacin and meropenem. Among the *E. coli* isolates from urine, 4/7 (57%) were susceptible to nitrofurantoin and gentamicin. Detailed resistance profiles for all organisms, including those with fewer than five isolates, are presented in Figure 3a. Among Gram-positive isolates, *S. epidermidis*, *E. faecium*, and *E. gallinarum* displayed multidrug resistance but retained susceptibility to glycopeptides and oxazolidinones (Figure 3b). *E. faecium* isolates from urine were resistant to tetracycline (88%, 7/8), benzylpenicillin (100%, 7/7), ampicillin (100%, 7/7), streptomycin (71%, 5/7), and levofloxacin (71%, 5/7) and susceptible to glycopeptides. *E. faecium* isolates from blood were resistant to tetracycline (67%, 4/6) and levofloxacin (83%,5/6) and susceptible to linezolid (100%, 5/5), teicoplanin (100%, 6/6), and vancomycin (100%, 5/5) (Figure 3b).

### 2.5. Multidrug- and Extensively Drug-Resistant Organisms

**Figure 4:**
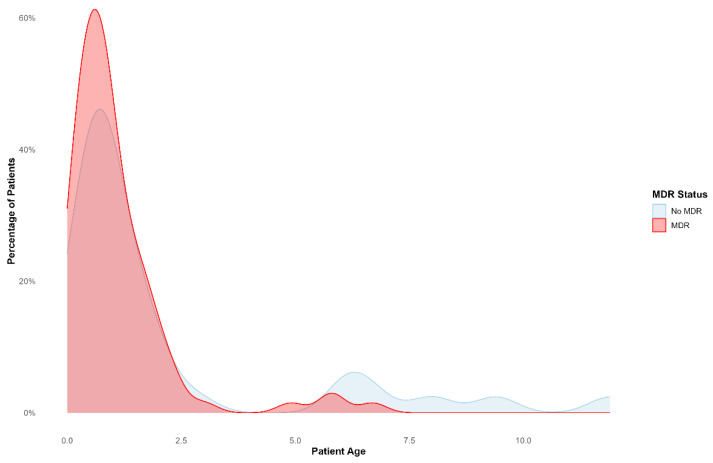
The figure illustrates the relationship between patient age and the presence of multidrug-resistant organism status (no MDRO or MDRO). The data reveals a peak in the younger age group for patients with MDRO, suggesting that age may be a risk factor for developing MDRO infections.

Among the patients tested, 84/111 (76%) were infected with MDROs, of which 25/84 (30%) were extensively drug-resistant organisms (XDR). The prevalence of MDROs among tested isolates was 92/128 (72%), of which 26/92 (28%) were XDR. Notably, 84/92 (91%) children under two years had infections caused by multidrug-resistant organisms, as shown in Figure 4. *K. pneumoniae* had the majority (20%, 18/92) of the MDROs, followed by *S. epidermidis* (15%,14/92), *E. faecium* (12%, 11/92), and *E. gallinarum* (11%, 10/92).

**Figure 5:**
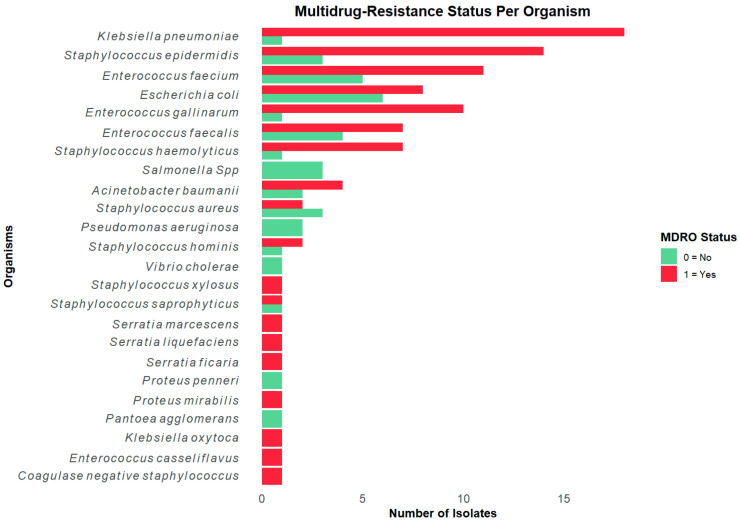
Distribution of multidrug-resistant organisms by bacterial species. The figure shows the stratification of multidrug-resistant organism (MDRO) status by organism isolated. *Klebsiella pneumoniae* and *Staphylococcus epidermidis* accounted for the highest proportions of MDRO isolates.

Stratifying per organism isolated, 18/19 (95%) *K. pneumoniae,* 10/11 (91%) *E. gallinarum,* 14/17 (82%) *S. epidermidis,* 11/16 (69%) *E. faecium,* and 8/14 (57%) *E. coli* were multidrug-resistant organisms, as shown in Figure 5.

**Figure 6:**
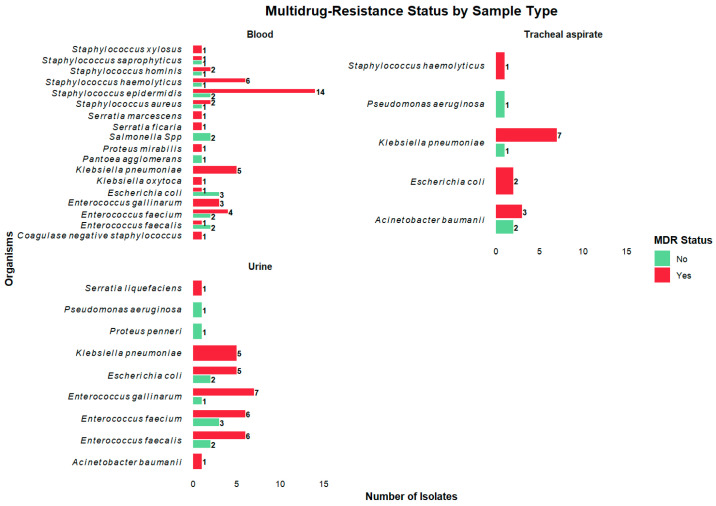
Distribution of multidrug-resistant (MDR) isolates from blood, tracheal aspirate, and urine samples. *Staphylococcus epidermidis* was the most common MDR isolate in blood samples, while *Klebsiella pneumoniae* was the predominant MDR pathogen in tracheal aspirate samples.

Stratifying MDROs by sample type, all *K. pneumoniae* isolates from blood (100%, 5/5) and urine (100%, 5/5) were multidrug resistant, while among isolates from tracheal aspirates, 7/9 (78%) were multidrug resistant. A total of 14/16 (88%) *S. epidermidis* isolates from blood were multidrug resistant. Comparing *E. coli* isolates from blood and urine, 5/7 (71%) from urine were multidrug resistant, while only 1/4 (25%) in blood were multidrug resistant (Figure 6).

Of the 26 XDRs isolated, 7/26 (27%) were *K. pneumoniae*, 5/26 (19%) *E. coli,* and 4/26 (15%) *A. baumannii*. We note 4 out of 6 (67%) *A. baumannii* isolated were XDR.

### 2.6. Association Between Drug-Resistant Infections and Mortality

Neither MDROs (OR = 0.69; 95% CI: 0.26–1.89; *p* = 0.464) nor XDR infections (odds ratio [OR] = 2.64; 95% CI: 0.95–7.36; *p* = 0.061) were statistically associated with patient mortality.

### 2.7. Factors Associated with Acquiring Multidrug-Resistant Organisms

Fisher’s Exact Test revealed a statistically significant association between age group and MDRO status (*p* = 0.015). Children aged under 5 had approximately 5.8 times higher odds of having an MDRO infection compared to those aged 5–12 years (OR: 5.84, 95% CI: 1.17–38.21). Patients with multiple hospital admissions had approximately 4.63 times higher odds of having an MDRO infection compared to those with a single admission (*p* = 0.037, 95% CI 1.03–42.99).

In the multivariable logistic regression analysis, two variables were significantly associated with MDRO infection. Patients with multiple hospital admissions had increased odds of having an MDRO, with an adjusted odds ratio (aOR) of 3.77 (95% CI: 1.06–20.34; *p* = 0.039). Additionally, increasing age was inversely associated with MDRO presence. The odds of MDRO infection decreased by 24% for every year increase in age (aOR = 0.76; 95% CI: 0.60–0.93; *p* = 0.006). Table 1 shows univariate logistics regression analysis.

The table presents logistic regression coefficients (β), odds ratios (OR), 95% confidence intervals (CI), and *p*-values for each independent variable. Variables with *p* < 0.2 were considered for inclusion in the multivariate Firth logistic regression model.

## 3. Discussion

This study presents an analysis of bacterial infections, diagnostic utilization and antimicrobial resistance among pediatric inpatients in a national referral hospital. The findings highlight critical gaps in diagnostic stewardship and the burden of AMR, especially MDROs, among hospitalized children in low-resource settings.

Gastroenteritis was the most frequently diagnosed infection in our study, a pattern that aligns with the existing literature emphasizing the continued predominance of enteric infections as a leading cause of pediatric morbidity and mortality across sub-Saharan Africa. This is particularly evident among children under five, where diarrheal diseases account for a significant proportion of hospital admissions and preventable deaths [24]. The high burden of gastroenteritis in this setting may reflect limited access to clean water, inadequate sanitation and suboptimal hygiene practices, all of which disproportionately affect children in low-resource settings [25,26]. Bacterial pneumonia and sepsis were also highly prevalent, reinforcing the findings from national surveillance and global health reports that identify these infections as major contributors to child mortality, especially in under-immunized and malnourished populations [27,28]. Pneumonia is the main infectious cause of death among children worldwide, and in African contexts, the risk is exacerbated by poor vaccine uptake, indoor air pollution, and delayed care-seeking behavior [29,30,31]. Similarly, the burden of pediatric sepsis, while often underrecognized due to diagnostic limitations, continues to be substantial in LMICs, with mortality rates significantly higher than those observed in high-income countries [32,33].

Despite the clinical burden of these infections, microbiological diagnostic utilization was low; only 40% of patients had cultures requested. Among the most common infections, i.e., gastroenteritis and pneumonia, fewer than half had cultures performed. This highlights a critical underutilization of diagnostics, which are foundational to effective antimicrobial stewardship. These findings align with challenges across LMICs, where inadequate laboratory infrastructure, limited personnel, and financial barriers restrict both access to and the clinical use of diagnostic testing [34]. This limited use of cultures impairs the ability to identify causative pathogens and determine their antibiotic susceptibility, leading to a heavy reliance on empirical therapy. In the absence of microbiological data, clinicians are often forced to prescribe broad-spectrum antibiotics with the inability to de-escalate, which may be inappropriate for the underlying infection and contribute to poor treatment outcomes, including delayed clinical recovery, prolonged hospital stays, and increased risk of mortality.

Additionally, this study reported a high no-growth rate (86%) in culture results. Several factors may explain this, including prior or empirical antibiotic use before sample collection, which can suppress bacterial growth. Suboptimal sampling practices (e.g., timing, volume, or contamination) and delayed processing can also compromise culture viability. These diagnostic inefficiencies contribute to inappropriate antibiotic use and underscore the critical role of timely, high-quality diagnostics in stewardship efforts. The WHO’s 2024 AMR targets—reducing AMR-associated mortality by 10% and ensuring 70% use of “Access” antibiotics—cannot be achieved without investments in laboratory systems and diagnostic stewardship [35]. Achieving these goals requires quality laboratory systems and diagnostic stewardship. The cerebrospinal fluid (CSF) culture positivity rate observed in this study was notably low (1%), substantially below the 12.9% reported in Leazer et al.’s study of term infants in high-resource settings [36]. This discrepancy may be explained by several factors intrinsic to LMIC settings, including challenges in sample collection, handling, and transport. CSF cultures are particularly sensitive to pre-analytical conditions such as delays in transport to the microbiology laboratory, improper temperature maintenance, or suboptimal aseptic technique—all of which can compromise organism viability and thus reduce culture yield. In our setting, such logistical and infrastructural constraints may have affected CSF diagnostic performance. Furthermore, prior antimicrobial exposure, a common occurrence in hospitalized children—can suppress bacterial growth in culture. Improvements in CSF sample handling and enhanced documentation of laboratory quality metrics are essential to strengthen the reliability of culture-based diagnostics in similar settings.

The high resistance burden in this study reinforces global concerns about the diminishing efficacy of first-line treatments in pediatric care [37]. The WHO Bacterial Priority Pathogens List has played a crucial role in shaping global policies, research and development initiatives, as well as investment decisions to address the most pressing threats posed by antibiotic-resistant bacteria [38]. *K. pneumoniae*, *E. coli*, and *A. baumanii*—all of which were isolated in this study—are ranked among the most critical global threats.

Similarly, in this study, *K. pneumoniae* and *E. coli* contribute to a large proportion of isolates. The three organisms demonstrated resistance to most antibiotics, classified as multidrug-resistant and XDR organisms. This burden is especially concerning in resource-constrained settings where the management of MDROs and XDR is hampered by limited workforce capacity, constrained supply chains for pediatric-appropriate formulations, and weak infection prevention and control systems.

*K. pneumoniae* isolates demonstrated a concerning resistance profile to several first-line and commonly used antibiotics, with notably high susceptibility retained only to a limited number of watch and reserve antibiotics. All isolates were resistant to ampicillin, ampicillin-sulbactam, cefazolin, cefuroxime, cefotaxime, ceftriaxone, and aztreonam. These findings are consistent with the well-documented intrinsic resistance of *K. pneumoniae* to ampicillin. However, the complete resistance to extended-spectrum cephalosporins and aztreonam suggests a broader resistance mechanism—likely ESBL-production or carbapenemase activity—both of which are increasingly prevalent in clinical Klebsiella isolates, particularly in LMICs [38,39,40], likely due to an overreliance on empirical third-generation cephalosporins for severe infections in hospitalized children in resource-limited settings. Amikacin remains a potent option in many African contexts, particularly because of its limited use compared to other aminoglycosides such as gentamicin, which often encounter higher resistance rates. However, amikacin use is constrained by the need for therapeutic drug monitoring and its potential for nephrotoxicity and ototoxicity—challenges in pediatric care, especially in resource-limited settings. These results suggest that carbapenems and aminoglycosides remain among the few effective therapeutic options for treating Klebsiella infections in this setting. The relatively high meropenem susceptibility is encouraging but also precarious, as carbapenems are often reserved for last-line treatment due to cost, limited availability, and the threat of emerging carbapenem-resistant Enterobacterales. *S. epidermidis*, a coagulase-negative staphylococcus (CoNS), showed full susceptibility to linezolid, teicoplanin, and vancomycin. These findings are consistent with other reports from LMICs where glycopeptides and oxazolidinones retain effectiveness against multidrug-resistant CoNS strains, particularly in neonatal intensive care units [41,42]. Though glycopeptides such as vancomycin have been considered as the first line of treatment for CoNS infections, empiric therapy with this drug can be reduced to avoid exposure to resistance [42]. Alternatively, in this study setting, clindamycin and tetracycline provide additional therapeutic options, especially where intravenous therapy is not feasible. Universal resistance to benzylpenicillin and elevated resistance to erythromycin and co-trimoxazole reflect widespread antimicrobial pressure and historical overuse of these agents in community and hospital settings. This pattern mirrors findings from other settings, where CoNS frequently shows resistance to β-lactams and macrolides [43,44,45], raising concerns about empirical treatment practices in pediatric populations. While *S. epidermidis* can be a pathogen in neonates and immunocompromised children, this finding must be interpreted with caution given potential contamination.

*E. faecium* isolates had complete susceptibility to teicoplanin and vancomycin and high susceptibility to linezolid and tigecycline. These findings are consistent with East African data showing that vancomycin-resistant enterococci remain relatively uncommon but are increasing [46,47]. Nevertheless, the complete resistance to ampicillin and benzylpenicillin and high-level tetracycline resistance are consistent with global trends of increasing multidrug resistance among enterococci [38,46,47,48]. Urinary isolates showed particularly high resistance to tetracycline, ampicillin, and streptomycin, posing a challenge in managing pediatric urinary tract infections, especially where glycopeptides are unavailable or costly. This underscores the need for stewardship and local susceptibility data to guide empiric therapy.

*E. coli* demonstrated substantial susceptibility to amikacin and meropenem and resistance to first-line β-lactams such as ampicillin and moderate susceptibility to nitrofurantoin, aligning with a study conducted in the same facility in 2015 [38]. However, the susceptibility of *E. coli* to amikacin seems to have decreased from 91–97% in said study to 83% in our study. This could be associated with the increased empiric prescription of amikacin. Compared to other studies, we report higher resistance to ampicillin and amoxicillin clavulanic [49]. Studies across LMICs have consistently reported widespread resistance of *E. coli* to third-generation cephalosporins due to the dissemination of extended-spectrum beta-lactamase (ESBL)-producing strains [50,51,52,53]. Multiple studies have also documented that third-generation cephalosporins—particularly ceftriaxone and ceftazidime—are among the most commonly prescribed antibiotics in both hospital and outpatient settings [54,55]. Consequently, there was high resistance for cefuroxime, cefotaxime, and ceftriaxone.

This study has several limitations. First, the definitions of MDR and XDR organisms were applied in accordance with international standards, specifically resistance to ≥3 antibiotic classes for MDR and resistance to all but one or two for XDR. However, because the panel of antibiotics tested varied across isolates, classifications were limited to the available test data per organism. As such, some resistance classifications may under- or overestimate the true MDR/XDR status. This also hindered comparison among isolates. Files lacking laboratory results where cultures or AST had been requested were excluded from this study. This may have introduced sampling bias as records missing results may reflect issues with access to diagnostics or record keeping. Second, AST was performed in only 17% of the total cohort, introducing the potential for selection bias. Given our selection of a national public referral hospital for this study, it is likely that AST orders were prioritized in severe or non-responsive infections, which may have led to an overrepresentation of resistance among those tested. Consequently, our estimates of MDRO and XDR prevalence cannot be generalized to all pediatric infections in the hospital or to less specialized or lower-level healthcare facilities. They nevertheless serve as a sentinel indicator of resistance burden within the tested subset; the low diagnostic utilization itself, coupled with high resistance in tested cases, highlights an urgent need for expanded access to microbiological diagnostics and real-time surveillance to better understand and manage pediatric AMR in similar settings. Third, the retrospective design may have introduced bias arising from the quality of microbiological processes, incomplete documentation or underreporting of prior antibiotic use, which could have influenced the culture positivity rates and AST results. We were also unable to differentiate between community-acquired and hospital-acquired infections, which may influence resistance patterns. Despite the significant associations identified using Fisher’s Exact Test, these findings should be interpreted with caution due to inherent limitations. Fisher’s Exact Test is most suitable for small sample sizes or sparse categorical data; however, it is also highly sensitive to sample distribution and does not account for potential confounders. The wide confidence intervals observed in our analysis, particularly for variables such as multiple hospital admissions, suggest imprecision in the estimates, likely due to the small number of events in some subgroups. This limitation reduces the generalizability of the results and may overestimate or underestimate the strength of associations. To confirm the association from the Fisher’s test, we conducted multivariate Firth logistic regression. To address these limitations and strengthen the evidence base, future multicenter studies with larger, prospectively collected datasets are recommended. Such studies should aim to include diverse healthcare settings to enhance the generalizability of findings and inform targeted interventions for antimicrobial stewardship across various levels of care.

## 4. Materials and Methods

### 4.1. Study Design and Setting

This was a retrospective cohort study conducted at the Kenyatta National Hospital (KNH) in Nairobi, Kenya. This study involved the abstraction of clinical and laboratory data from paper-based medical records of pediatric patients aged 0–12 years admitted with urinary tract infections, gastroenteritis, bacterial meningitis, bacterial sepsis, wound infection, bacterial pneumonia, tonsilitis, pharyngitis, and tracheitis between January 2017 and December 2021.

### 4.2. Study Population and Eligibility Criteria

All files of children admitted with bacterial infections of interest were retrieved using the specific International Statistical Classification of Diseases and Related Health Problems, 10th Revision (ICD-10) codes corresponding to these conditions, maintained by the hospital records department. Records were excluded if they fell outside the study period, did not match the defined infection categories, or were missing essential documentation. Specifically, files lacking laboratory results (where cultures or antimicrobial susceptibility testing (AST) had been requested), treatment sheets, demographic data, patient outcomes, or admission history were excluded.

### 4.3. Data Collection Procedures

A structured questionnaire was developed in REDCap and deployed on mobile devices to guide the data abstraction process. The tool was pretested before data collection. Information collected included patient demographics (age, sex), admission and discharge dates, referral status, diagnosis, comorbidities, sample collection (type and date), laboratory investigations (culture, AST), treatment details, surgical history, previous admissions, and clinical outcomes.

Microbiological data were abstracted from patient medical records and laboratory reports. This included information on whether microbiological culture or AST was performed, the type of sample collected, and the identity and susceptibility profile of any isolated organism. No laboratory procedures were conducted or verified by the study team; all data reflect routine clinical diagnostics as recorded in the patient files.

Only one isolate per organism per patient admission was included to avoid duplication. A multidrug-resistant organism (MDRO) was defined as one resistant to at least one agent in three or more antimicrobial classes, and extensively drug-resistant organisms (XDRs) were defined as those resistant to all but one or two antimicrobial classes.

### 4.4. Data Analysis

Data cleaning and analysis were conducted using R software version 4.4.2 [56]. Descriptive analysis was conducted for key variables, including infection counts, diagnostic testing (culture and AST), and resistance outcomes (MDRO and XDR classifications).

In the antibiotic resistance section, we analyzed one isolate if the same organism was found in two samples of the same patient during the same admission. We prioritized the most common samples, which included blood, stool, urine, CSF, and tracheal aspirate. In some instances, when the panel of antibiotics tested varied across isolates, we have reported the data as they were tested and presented in the patient records.

Given the small sample size in our dataset, which rendered the Chi-square approximation of associations between categorical variables and MDRO status potentially inaccurate, we employed Fisher’s Exact Test, which is more reliable for small sample sizes and sparse data. We determined whether there was a statistically significant association between age group, repeat admission, and MDRO status. A *p*-value < 0.05 was considered statistically significant.

Univariate logistic regression was used to explore associations between MDRO and patient-level characteristics, including age, referral status, comorbidities, surgical history, prior admissions, length of hospital stay, and presence of multiple infections. Variables with a *p*-value less than 0.20 were selected for multivariable modeling. Due to the presence of small cell counts and potential separation in the data, particularly in variables with rare events, we employed Firth multivariate logistic regression analysis. This method uses penalized maximum likelihood estimation to reduce small-sample bias and to handle issues of quasi-complete or complete separation, which can lead to unreliable or infinite estimates in traditional logistic regression. Adjusted odds ratios (aORs) with 95% confidence intervals and corresponding *p*-values were reported. Model fit was evaluated using the penalized likelihood ratio test.

Logistic regression was used to assess the association between MDRO and XDR infections and patient outcomes. Odds ratios (ORs) with 95% confidence intervals (CIs) were reported. Given the small sample size, the results were interpreted cautiously due to limited statistical power.

## 5. Conclusions

This study highlights a critical intersection between underutilized diagnostic resources and a high burden of antibiotic resistance among hospitalized children in a national referral hospital in Kenya. The prevalence of MDROs, particularly *K. pneumoniae*, *E. coli*, and *Enterococcus* species, presents serious challenges for empirical therapy, infection control and clinical outcomes. Resistance to commonly used first-line antibiotics was widespread, while susceptibility to last-line antibiotics such as meropenem, vancomycin, and linezolid was largely observed. Although these drugs remain effective in many cases, their limited availability, cost, and potential toxicity reinforce the need for judicious, diagnostics-guided use. While empirical treatment remains necessary in many LMIC settings, particularly where diagnostic capacity is limited or delayed, it should be complemented by efforts to expand utilization and access to timely, high-quality microbiological testing to support targeted therapy and enhance antimicrobial stewardship. Strengthening infection prevention and control measures, especially for high-risk groups such as infants and frequently hospitalized children, is essential. National strategies should prioritize laboratory infrastructure, ongoing provider training, and the integration of pediatric resistance surveillance into broader AMR monitoring frameworks to safeguard antibiotic efficacy and improve child health outcomes.

## Figures and Tables

**Table 1:** Univariate logistic regression analysis of factors associated with multidrug-resistant organism (MDRO) infections among hospitalized children.

Variable	Coefficient	Odds Ratio	95% CI	*p*-Value (<0.2)
Length of stay	0.026	1.03	0.99–1.08	0.214
Referral status	0.310	1.36	0.64–2.95	0.425
Age	−0.299	0.74	0.58–0.91	0.008
Multiple infections	−0.310	0.73	0.34–1.57	0.425
Comorbidity	0.593	1.81	0.81–4.01	0.144
Surgery	0.041	1.04	0.28–4.97	0.953
Multiple admissions	1.540	4.47	1.27–30.25	0.045

## Data Availability

The data presented in this study are openly available in Open Science Framework: DOI 10.17605/OSF.IO/Z76N9 and DOI 10.17605/OSF.IO/ADR7S. These datasets are available under CC0 1.0 Universal license.

## Supplementary Materials

The following supporting information can be downloaded at: https://www.mdpi.com/article/10.3390/antibiotics14090872/s1, Figure S1: Proportion of culture and sensitivity test requests per infection type. The bar chart shows the proportion of pediatric patients for whom culture and sensitivity testing was requested, stratified by infection type. Each bar represents the relative frequency of test requests (Yes) versus no test requested (No) among all cases of each infection. Figure S2: Temporal distribution of culture requests among hospitalized children from 2017 to 2021. The stacked bar graph shows the annual number of cases in which bacterial cultures were requested (Yes) versus not requested (No). 2021 and 2018 had the highest numbers of cultures requested at 49.39% and 44.80% respectively.
