# Supplementary material for: Diagnostic Underuse and Antimicrobial Resistance Patterns Among Hospitalized Children in a National Referral Hospital in Kenya: A Five-Year Retrospective Study
Source: Antibiotics (Basel). 2025 Aug 29;14(9):872. doi: 10.3390/antibiotics14090872 (PMC12466545; doi:10.3390/antibiotics14090872)
Supplement: Supplementary file 1 [file antibiotics-14-00872-s001.zip › antibiotics-3774693-supplementary.pdf]

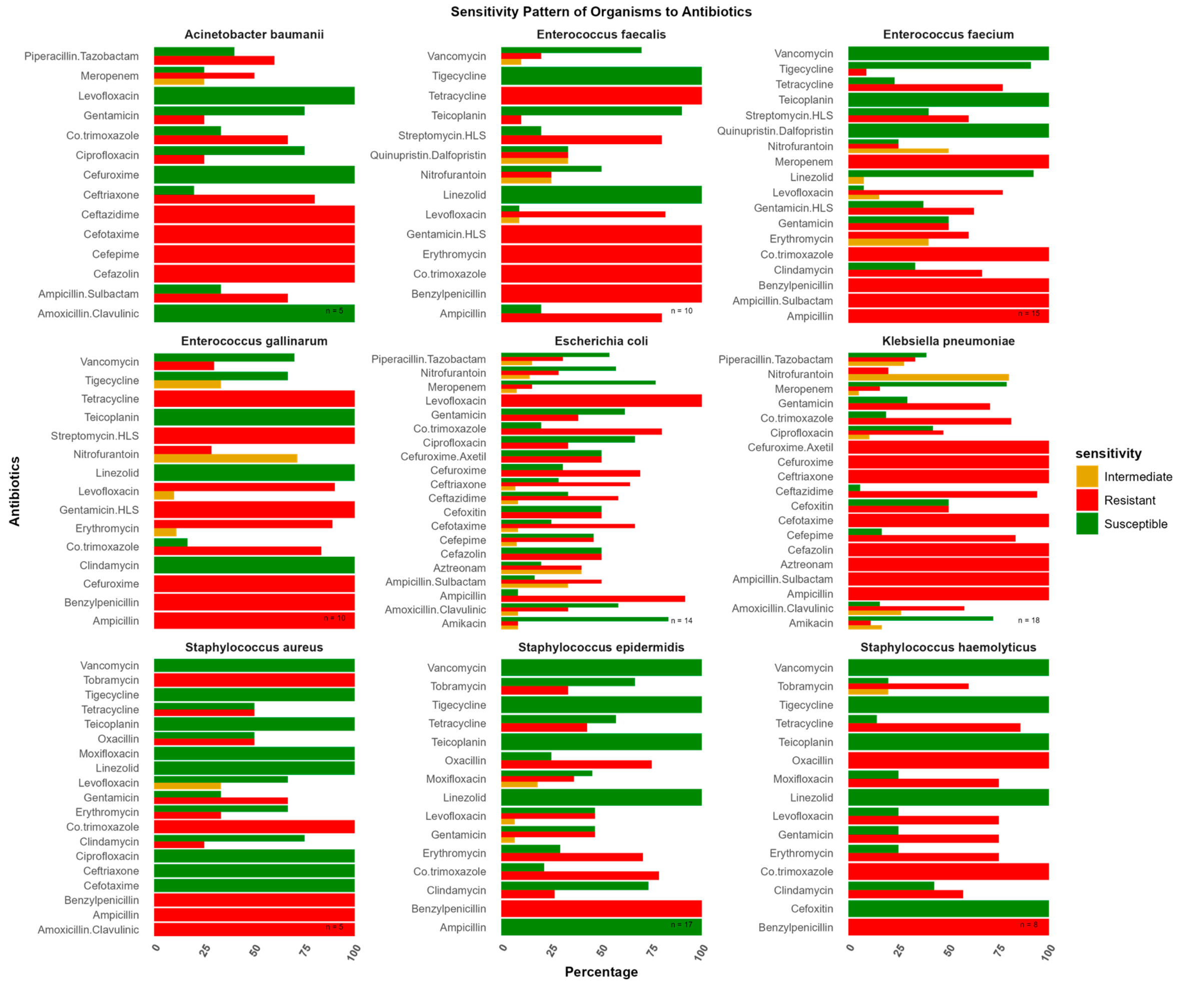

Figure S1. Proportion of culture and sensitivity test requests per infection type. The bar chart shows the pro-portion of pediatric patients for whom culture and sensitivity testing was requested, stratified by infection type. Each bar represents the relative frequency of test requests (Yes) versus no test re-quested (No) among all cases of each infection.

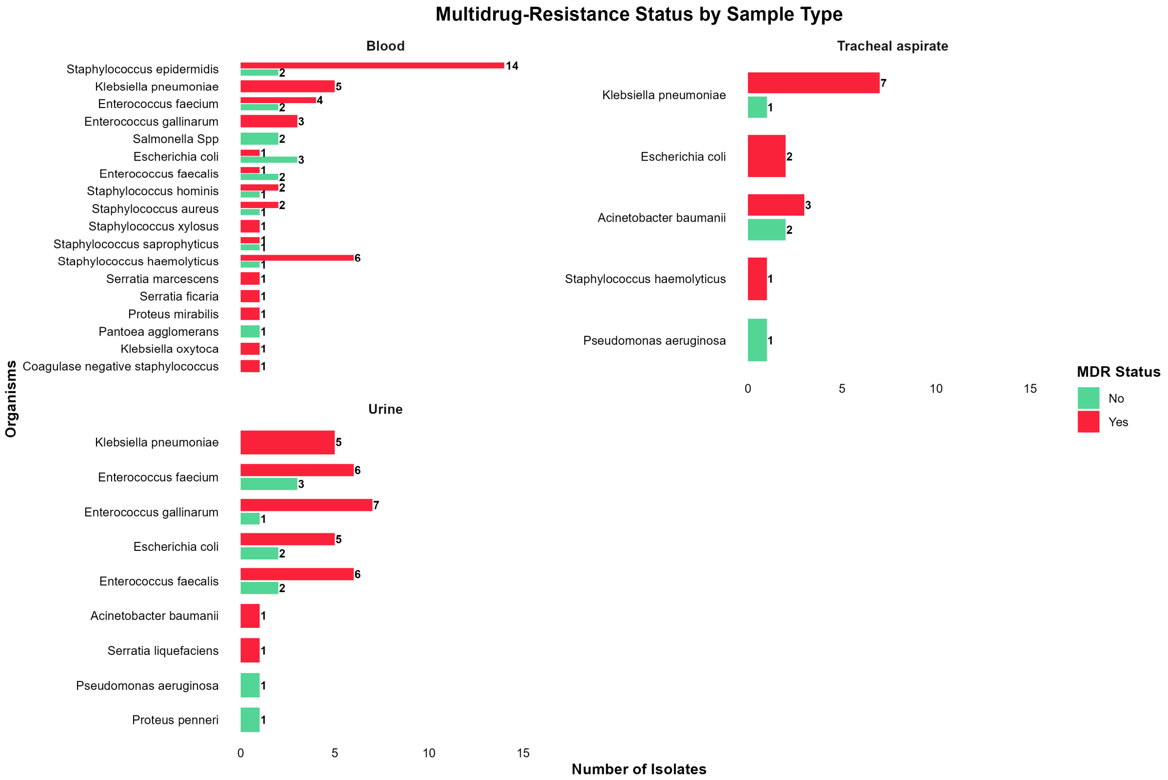

Figure S2. Temporal distribution of culture requests among hospitalized children from 2017 to 2021. The stacked bar graph shows the annual number of cases in which bacterial cultures were requested (Yes) versus not requested (No). 2021 and 2018 had the highest numbers of cultures requested at 49.39% and 44.80% respectively.
